# Low-Cost Distributed Optical Waveguide Shape Sensor Based on WTDM Applied in Bionics

**DOI:** 10.3390/s23177334

**Published:** 2023-08-22

**Authors:** Kai Sun, Zhenhua Wang, Qimeng Liu, Hao Chen, Weicheng Cui

**Affiliations:** 1 Zhejiang University-Westlake University Joint Training, Zhejiang University, Hangzhou 310027, China; 2 Key Laboratory of Coastal Environment and Resources of Zhejiang Province (KLCER), School of Engineering, Westlake University, Hangzhou 310030, China

**Keywords:** optical waveguide, WTDM, distributed shape sensor, bionics

## Abstract

Bionic robotics, driven by advancements in artificial intelligence, new materials, and manufacturing technologies, is attracting significant attention from research and industry communities seeking breakthroughs. One of the key technologies for achieving a breakthrough in robotics is flexible sensors. This paper presents a novel approach based on wavelength and time division multiplexing (WTDM) for distributed optical waveguide shape sensing. Structurally designed optical waveguides based on color filter blocks validate the proposed approach through a cost-effective experimental setup. During data collection, it combines optical waveguide transmission loss and the way of controlling the color and intensity of the light source and detecting color and intensity variations for modeling. An artificial neural network is employed to model and demodulate a data-driven optical waveguide shape sensor. As a result, the correlation coefficient between the predicted and real bending angles reaches 0.9134 within 100 s. To show the parsing performance of the model more intuitively, a confidence accuracy curve is introduced to describe the accuracy of the data-driven model at last.

## 1. Introduction

Under the background of rapid development of artificial intelligence, new materials and manufacturing technologies, bionic robots have emerged as a popular and promising research topic [1,2,3,4]. Flexible materials and sensors play a crucial role in the design and manufacturing of bionic robots, as they enable the creation of non-destructive, flexible robots with perception, bridging the gap between organisms and machines [5,6]. For instance, in the field of marine biology, biologists prefer using flexible manipulators for sampling, observing, and conducting experiments on delicate organisms [7]. However, standard communication fibers are not flexible or stretchable, and their complex optical paths, high-precision, and bulky demodulation equipment take up high cost [8]. Though researchers have tried to miniaturize the devices, the balance between precision and size remains a problem [9]. Optical waveguides made of transparent flexible materials, such as polyimide (PI), polydimethylsiloxane (PDMS), polyethylene terephthalate (PET), etc., whose flexibility is similar or identical to those flexible manipulators, offer great advantages when used for sensing [10,11,12]. It seldom affects the flexibility of the manipulators [13]. However, optical waveguides are often used as integrated sensors based on light attenuation when bending, which leads to a low level of sensing capability [14,15,16]. In 2016, Zhao et al., realized the function of bending and sensing surface roughness with bionic fingers by measuring the light intensity variations [17]. Despite the realization of the above functions, the sensing capability is completely dependent on the number of waveguides. In 2020, Kim et al., used machine learning to integrate optical, ionic liquid, and conductive fabric sensing methods to achieve recognition of bending angle and direction [18]. Flexible sensors that incorporate three sensing modalities increase the complexity of the manufacturing process as well as signal processing. In the same year, Bai et al., combined wavelength division multiplexing (WDM) principles, using color filter blocks to absorb part of the color light when white light passes through and based on the RGB color sensor, it achieves qualitative sensing of the strain location on the optical waveguide and bending angles [19,20]. Based on this work, Sun et al., quantitatively implemented quasi-distributed optical waveguide shape sensing based on a data-driven model with a certain degree of accuracy [5]. However, models based on neural networks require abundant data to improve the accuracy of the model.

In this paper, a data-driven distributed sensing approach based on wavelength and time division multiplexing (WTDM) is proposed. This method combines optical waveguide transmission loss and the way of controlling the color and intensity of the light source to theoretically verify the feasibility of localizing the bending at multiple locations based on intensity variations. In practice, due to the accuracy limitations of the sensor components and the disturbance from external environmental factors, the waveguides are compensated through the incorporation of dyed color filter blocks. The WTDM method is used for data acquisition because richer sensing data can be obtained by changing the intensity and color of the light source in a short period of time. Finally, a machine learning approach is used to model the sensing signals and predict the bending angle of a bionic joint targeting a 4-degree-of-freedom joint. The WTDM-based signal acquisition method enhances the data capacity of the optical waveguide sensor, which leads to the improvement of the accuracy of the neural network-based optical waveguide sensor.

## 2. Principles and Methods

### 2.1. Sensing Principles

**Figure 1:**
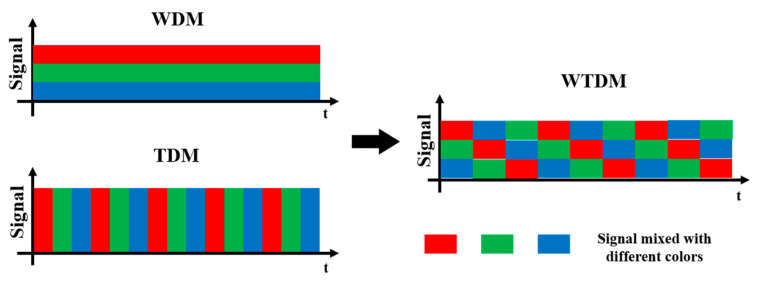
Principles of wavelength and time division multiplexing.

WDM and Time Division Multiplexing (TDM) are two pivotal techniques utilized in telecommunications to enhance the efficiency and capacity of data transmission over a single communication channel [21]. WDM facilitates the simultaneous transmission of multiple signals over a single optical fiber by employing distinct wavelengths or colors of light. As depicted in Figure 1, each signal is allocated a unique wavelength and merged with other signals to form a composite signal that can be transmitted through the optical fiber. At the receiving end, demodulation devices are employed to separate the composite signal into its constituent signals, enabling the independent extraction and processing of individual signals. On the other hand, TDM assigns discrete time slots to different signals, enabling the sharing of a single communication channel by multiple signals. In TDM, each input signal is divided into fixed time intervals or slots, which are subsequently combined sequentially to construct a composite signal. The composite signal is then transmitted over the channel, and at the receiving end, the original signals are reconstructed by demultiplexing the composite signal based on the assigned time slots. In this paper, we propose a combination of WDM and TDM, referred to as WTDM, as illustrated in Figure 1. This hybrid approach enables an increased capacity for sensing data collection and transmission.

**Figure 2:**
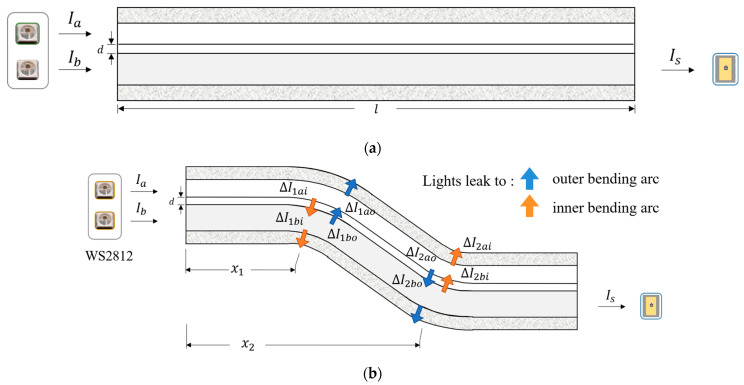
Intensity change when waveguides are (**a**) straight (**b**) bent at $x_{1}$ and $x_{2}$.

The complex refractive index of the light propagating medium can be expressed as $\tilde{n}=n+i\kappa$, where n is the real part of the refractive index, κ is the imaginary part of the refractive index, and the relationship with the absorption coefficient of light can be expressed as α=4κπ/λ [22]. The intensity of light after propagating x distance in the medium can be expressed as [23]:

(1) $${I=I}_{0}e^{-\alpha x}$$

where, $I_{0}$ is the initial light intensity. Combining the loss of light propagation in the medium and the principle of wavelength division/time division multiplexing, we present a distributed optical waveguide sensing approach with adjustable LED light source. As shown in Figure 2a, it is composed of two waveguides, and the left end of each waveguide is equipped with a color and intensity an adjustable LED light source WS2812, whereas the other end of the main waveguide is stuck to an RGB color detector TCS3472. The LED light source sends lights in different intensities and colors (wavelengths) almost at the same time so that multiple equations can be formulated using the difference of the light source. Combined with the output intensity and color, the equations can be solved, and sensing information can be obtained. As shown in Figure 2b, we take the bending of the waveguide at $x_{1}$ and $x_{2}$ as an example.

Figure 2a shows the optical waveguide is in a straight state. When only the light source $I_{b}$ of the main waveguide is turned on, the light intensity received by the color detector TCS3472 can be expressed as:

(2) $$I_{s0}=I_{0}$$

**Figure 3:**
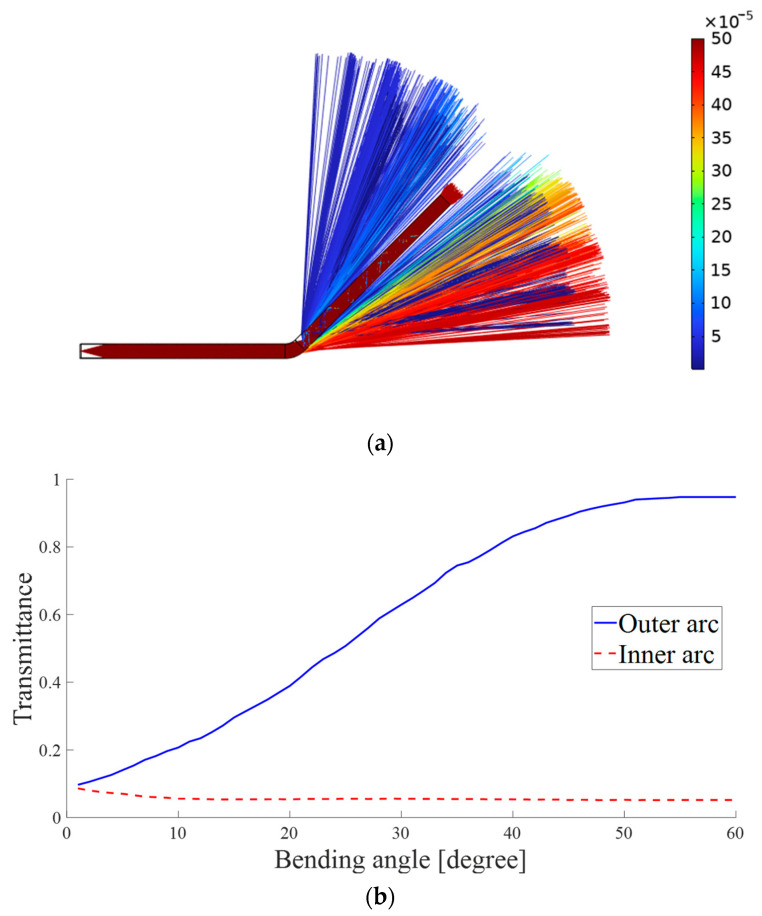
(**a**) Light path and intensity when the waveguide bent. (**b**) Variation of light leakage percentage with bending angle.

Figure 2b shows the optical waveguide is bent both at $x_{1}$ and $x_{2}$. The optical intensity in the waveguide at $x_{1}$ and $x_{2}$ are $I_{1}$ and $I_{2}$, respectively. Besides, light rays that escape from outside the bending arc are significantly more than light rays that escape from inside the bending arc. With the geometrical optics module of COMSOL, we simulated the state of the waveguide as it bent and tracked the light rays. Figure 3a shows the ray path when the waveguide is bent. Figure 3b shows the amount of light transmitted from the outside and inside of the bending arc, respectively, when the waveguide is bent from zero to 60 degrees. $∆I_{i}$ and $∆I_{o}$ are used to express the leaked intensity from the inside and outside of the bending arc at the same location individually. Assuming that the proportion of light rays escaping from the outside bending arc in the waveguide is β and the proportion of light rays escaping inside the bending arc is γ, the light intensity changes at the $x_{1}$ bend can be written as follows:

(3) $$\left\{\begin{array}{c} {\Delta I}_{1ao}=\beta I_{a}e^{-\alpha_{0}x_{1}}\\ {\Delta I}_{1bo}=\beta I_{b}e^{-\alpha_{0}x_{1}} \end{array}\right.$$

(4) $$\left\{\begin{array}{c} {\Delta I}_{1ai}=\gamma I_{a}e^{-\alpha_{0}x_{1}}\\ {\Delta I}_{1bi}=\gamma I_{b}e^{-\alpha_{0}x_{1}} \end{array}\right.$$

where, $\alpha_{0}$ is the absorption coefficient of the waveguide material. Therefore, the intensity of light escaping from the waveguide at the $x_{2}$ bend is:

(5) $$\left\{\begin{array}{c} {\Delta I}_{2ao}=\beta \left(I_{a}e^{-\alpha_{0}x_{1}}-{\Delta I}_{1ao}-{\Delta I}_{1ai}+{\Delta I}_{1bo}\right)e^{-\alpha_{0}{(x}_{2}-x_{1})}\\ {\Delta I}_{2bo}=\beta \left(I_{b}e^{-\alpha_{0}x_{1}}-{\Delta I}_{1bo}-{\Delta I}_{1bi}+{\Delta I}_{1ai}\right)e^{-\alpha_{0}{(x}_{2}-x_{1})} \end{array}\right.$$

(6) $$\left\{\begin{array}{c} {\Delta I}_{2ai}=\gamma \left(I_{a}e^{-\alpha_{0}x_{1}}-{\Delta I}_{1ao}-{\Delta I}_{1ai}+{\Delta I}_{1bo}\right)e^{-\alpha_{0}\left(x_{2}-x_{1}\right)}\\ {\Delta I}_{2bi}=\gamma \left(I_{b}e^{-\alpha_{0}x_{1}}-{\Delta I}_{1bo}-{\Delta I}_{1bi}+{\Delta I}_{1ai}\right)e^{-\alpha_{0}\left(x_{2}-x_{1}\right)} \end{array}\right.$$

After turning on all the two groups of group light sources, the color detector can measure the light intensity as $I_{s1}$ to obtain a set of equations as:

(7) $$I_{s1}=I_{0}-{\Delta I}_{1bo}-{\Delta I}_{1bi}+{\Delta I}_{1ai}+{\Delta I}_{2ao}-{\Delta I}_{2bi}-{\Delta I}_{2b0}$$

Substituting Equations (2)–(6) into Equation (7), we get:

(8) $$I_{s1}=I_{s0}+\gamma I_{a}e^{-\alpha_{0}x_{1}}+\left(\beta -\beta^{2}-2\beta \gamma -\gamma^{2}\right)I_{a}e^{-\alpha_{0}x_{2}}-\left(\beta +\gamma \right)I_{b}e^{-\alpha_{0}x_{1}}+\left(2\beta^{2}+2\gamma \beta -\beta -\gamma +\gamma^{2}\right)I_{b}e^{-\alpha_{0}x_{2}}$$

In the above equations, $x_{1}$ and $x_{2}$ are unknown quantities to be solved, while the light ratios β and γ escaping at the bend are unknown but definite values for a given scenario. The absorption coefficient $\alpha_{0}$ is a constant determined by the material of the waveguide, and $I_{s0}$, $I_{s1}$, $I_{a}$, and $I_{b}$ can be obtained from the tunable light source and the color detector, respectively. In summary, there are only two unknowns in Equation (8).

Combined with the simulation results in Figure 3 for the transmission inside and outside the bending arc when the waveguide is bent [24], it can be found that the value of γ is very small, almost zero, then Equation (8) can be simplified to:

(9) $$I_{s1}\approx I_{s0}+\left(\beta -\beta^{2}\right)I_{a}e^{-\alpha_{0}x_{2}}-\beta I_{b}e^{-\alpha_{0}x_{1}}+\left(2\beta^{2}-\beta \right)I_{b}e^{-\alpha_{0}x_{2}}$$

Based on the idea of WTDM principle, the input light intensity, i.e., the values of $I_{a}$ and $I_{b}$ are tunable, while the corresponding values of $I_{s0}$ and $I_{s1}$ can be obtained through the detectors at the end of the waveguide. A set of equations can be obtained by multiple sets of input and detector values with different light intensities and colors. Sufficient information is able to easily find out multiple bending positions of the waveguide as well as for β values.

### 2.2. Sensing Methods

**Figure 4:**
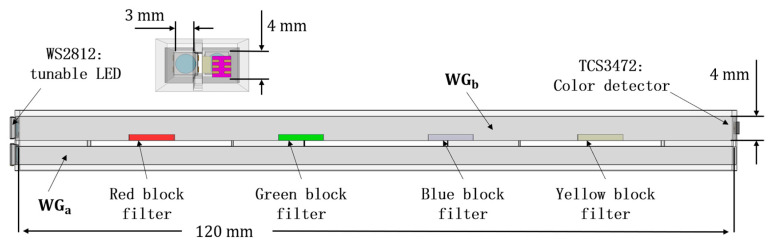
Optical waveguide shape sensor with color filter block.

The principle of distributed flexible optical waveguide sensor in ideal state is analyzed and derived in detail previously. However, during the practical experimental process, it is difficult to achieve precise verification of fully distributed sensing due to the low collimation of LED light sources, the optical property differences of the flexible light-guiding material, and the accuracy of the detector. Therefore, we designed a quasi-distributed shape sensor based on WTDM by embedding color filter blocks in the flexible optical waveguide. The length of the waveguides is 120 mm. The sectional dimensions are 4×4 mm and 3×4 mm, respectively. Besides, the thickness of color filter block is 1 mm, which is integrated in ${WG}_{b}$. The color filter blocks have strong selective effects on light colors and can effectively enhance the sensitivity of the waveguide sensor. As shown in Figure 4, the entire structure of the flexible optical waveguide sensor is designed asymmetrically and manufactured by pouring through molds. It has two waveguides made of PDMS, placed in parallel with a 1 mm distance. The outer cladding of the waveguides is made of Dragon Skin 10. It possesses a low refractive index, reducing light leakage based on the principle of total internal reflection. The distance between the two waveguides is used to prevent the light from coupling with each other to disturb the transmission when the waveguides are in a straight state. According to the comprehensive simulation results and test experience, the middle spacing vacancy can also ensure that the light from waveguide ${WG}_{a}$ can be coupled into the main waveguide ${WG}_{b}$ easily and finally detected by the color detector at the end of ${WG}_{b}$, when the waveguides bend close to each other or even in contact. In addition, two tunable LEDs, WS2812 and a color detector, TCS3472, for detecting the output color intensities of the waveguide ${WG}_{b}$ are placed at one end of the waveguide sensor. The tunable light sources WS2812 are plain LEDs. They emit diffuse light rays. To make the lights more directional, lenses are configured between the waveguides and the light sources for focusing the light and reducing stray light rays. Moreover, four different color filter blocks are embedded in the four positions of detecting bending in the waveguide ${WG}_{b}$, including red, green, blue, and yellow. When the optical waveguide sensor is bent, some light rays from the waveguide ${WG}_{a}$ will escape and then enter waveguide ${WG}_{b}$ through filter blocks, and only one color of light is allowed to enter, while other colors of light will be largely absorbed by the color filter blocks. At the same time, the LED lights in the waveguide ${WG}_{b}$ will also escape due to the bending of the waveguide so that the whole waveguide sensor can produce variations of color difference when bending. In addition, the light rays escaping from the waveguide ${WG}_{a}$ to the direction of waveguide ${WG}_{b}$ when the waveguide is bent in both positive and negative directions are obviously different, as shown in Figure 3, which makes it possible for the sensor to determine the direction of the waveguide bending.

In order to minimize the interference of the external environment with the experiments, all the tests were carried out at room temperature, and the experimental setups were covered in a blackout hood during experiments. In this experiment, the output color and intensity of the two LED light sources can be programmed by combining the WTDM principles to output different color lights to obtain multiple sets of sensing information. We used four different colors of light as inputs for waveguide ${WG}_{a}$, namely, red, green, blue, and yellow light. These four colors correspond to four color filter blocks. Meanwhile, for different input light intensities and colors, the color detector collects the output at the end of the waveguide ${WG}_{b}$. The waveguides are driven by the servo to bend between ±40 degrees, and the positive direction is recorded when the waveguide sensor as a whole bends to ${WG}_{a}$, and the negative direction is recorded when it bends to the other side. 

## 3. Results and Analysis

### 3.1. Single Bending Sensing

The principle of WTDM is to transmit sensing signals in time division with different colors (wavelengths). For each bending state of the waveguide sensor in this experiment, LEDs emit four different color lights, including red, green, blue, and yellow lights so that the color detector at the end of the waveguide ${WG}_{b}$ can collect four sets of sensing data, respectively. Besides, the waveguide ${WG}_{b}$ has also its background light in order to indicate the bending angles because the variations in light intensity and the angle of waveguide bending are closely related. Compared to the light in waveguide ${WG}_{a}$, the background light in waveguide ${WG}_{b}$ is relatively weak, but it is the sum of the same intensity of the RGB lights.

When the optical waveguide sensor is bent, the deformation caused by the squeezing of the flexible optical waveguide makes the gap between the waveguides decrease, or even contact occurs between them so that the light can be better coupled from the waveguide ${WG}_{a}$ to the waveguide ${WG}_{b}$. However, due to the filtering color block, the color light of the same color as the filtering block can pass through smoothly, but the intensity of different color light will be attenuated drastically due to the absorption effect.

**Figure 5:**
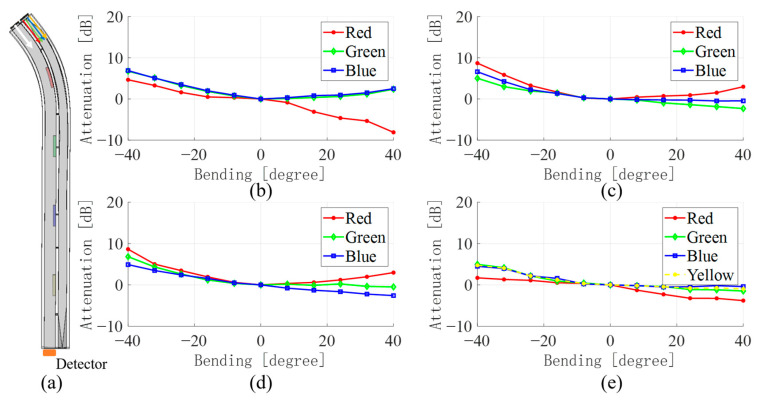
(**a**) 3D model of waveguide bending at the red filter block (**b**) ${WG}_{a}$ change in light intensity when red, (**c**) green, (**d**) blue, (**e**) yellow lights emit.

**Figure 6:**
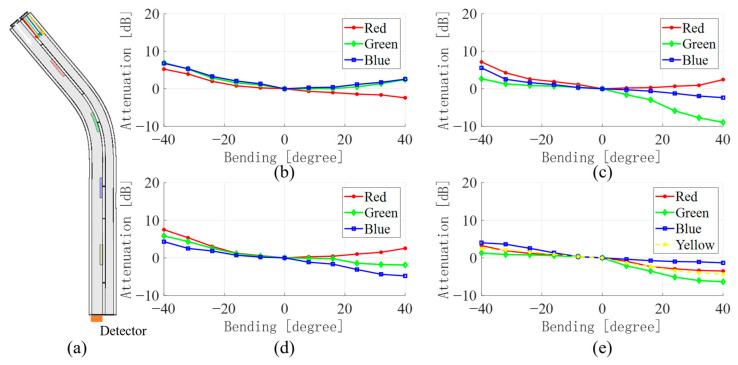
(**a**) 3D model of waveguide bending at the green filter block (**b**) ${WG}_{a}$ change in light intensity when red, (**c**) green, (**d**) blue, (**e**) yellow lights emit.

**Figure 7:**
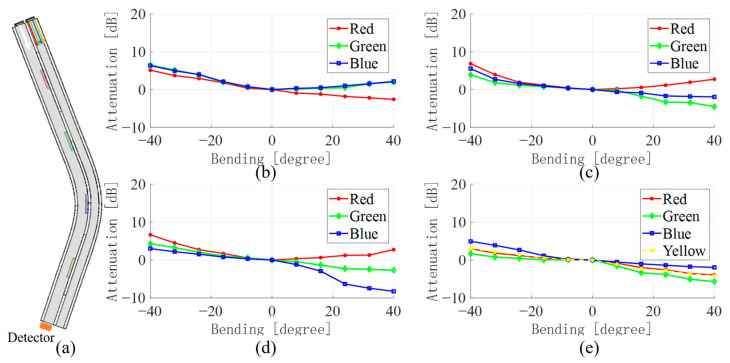
(**a**) Bending model of waveguide at the red filter block (**b**) ${WG}_{a}$ change in light intensity when red, (**c**) green, (**d**) blue, (**e**) yellow light is input.

**Figure 8:**
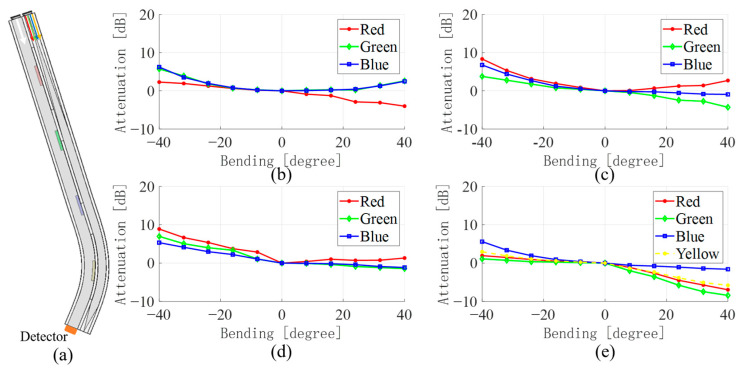
(**a**) Bending model of waveguide at the red filter block (**b**) ${WG}_{a}$ change in light intensity when red, (**c**) green, (**d**) blue, (**e**) yellow light is input.

Figure 5, Figure 6, Figure 7 and Figure 8 show the average values of RGB attenuations when the waveguide is bent from −40 degrees to 40 degrees at the four positions with different color filter blocks. The 3D model on the left shows the bending of the waveguide at −40 degrees at the corresponding filter block position. 

Firstly, the waveguide sensor bends at the red filter block, and the experimental results are shown in Figure 5. Figure 5a illustrates the bending of the waveguide sensor with a 3D model when the waveguides bend to −40 degrees at the red filter block. The four graphs in Figure 5b–e correspond to the relationship between the light attenuation of the four emitted lights and the bending angle of the waveguides. As the waveguides bend, light rays escape from both waveguides ${WG}_{a}$ and ${WG}_{b}$, which results in an increase in intensity attenuation. At the same time, the lights coupling from ${WG}_{a}$ to ${WG}_{b}$ should also be taken into consideration. When the waveguides bend to the negative direction, only a very small number of light rays can be coupled into ${WG}_{b}$ from the inside bending arc because most light rays escape from the outside bending arc. However, when the waveguides bend to the positive direction at the color filter block, light rays pass through the color filter block and couple into ${WG}_{b}$. Due to absorption effects, the light intensity of the corresponding color increases, while it has less effect on the other light colors transmitted by the waveguide ${WG}_{b}$.

Figure 5b shows the situation that the RGB lights are transmitted in waveguide ${WG}_{b}$ as background light and red-light rays are emitted into waveguide ${WG}_{a}$. The light intensity when the waveguide is in a straight state is used as the reference. The average light intensity attenuations of the waveguide in bending between −40 and 40 degrees are calculated and shown in Figure 5b–e. In general, Figure 5b displays that the red-light intensity attenuation goes down when waveguides bend to 40 degrees. That is because more red-light rays couple into ${WG}_{b}$ through a red color filter block. When waveguides bend to the negative direction, the light intensity attenuation does not decrease but is weaker than green and blue lights. The reason is as mentioned above. Light rays do not couple into ${WG}_{b}$ from the inside bending arc. However, some red-light rays still enter ${WG}_{b}$ during transmission because the LED lights are not directional enough. Parts of red-light rays escape from ${WG}_{a}$ through the spacing vacancy between ${WG}_{a}$ and ${WG}_{b}$ and then couple into ${WG}_{b}$ occasionally.

Figure 5c,d shows the attenuation variations of green and blue lights emitted in waveguides ${WG}_{a}$, respectively. Unlike red color lights, green and blue lights are not as pure as red lights. They contain each other’s color lights. Table 1 shows the RGB trichromatic proportion of the color light produced by the WS2812 LED light source. Lights in waveguide ${WG}_{b}$ escape as the waveguides bend, while lights from waveguide ${WG}_{a}$ are absorbed by color filter block partly. Even though when waveguides bend to the positive direction, stray lights will also couple into the waveguide ${WG}_{b}$ somehow, as mentioned previously.

When the LED at the initial end of the waveguide ${WG}_{a}$ is tuned to yellow light, the results are shown in Figure 5e. Since LEDs have only RGB color lights, yellow light is produced by superimposing red and green lights. Therefore, the yellow dashed line is drawn by calculating the red and green light attenuation in Figure 5e. Due to the yellow (red + green) light in waveguide ${WG}_{a}$ and yellow color filter block in waveguide ${WG}_{b}$, some red and green light will couple into waveguide ${WG}_{b}$ when sensor bends to the positive direction, which reduces the attenuation.

In addition, the results for the bends at the green filter block, the blue filter block, and the yellow filter block are shown in Figure 6, Figure 7 and Figure 8, respectively. Similar to the bend at the red filter block, as in Figure 5, by comparing the light intensity attenuation of the four images, the location of the waveguide bend can be quickly determined. The light passing through the filter color block into the waveguide ${WG}_{b}$ will result in a significant increase in the corresponding light intensity due to the absorption effect, as in Figure 6c and Figure 7d. However, the bend at the yellow filter block is a little different. As Table 1 shows, yellow light contains all the three-color lights, and the difference among the three colors is not too much. It results in a less pronounced attenuation of the yellow light in Figure 8e than that located at the red, green, and blue filter blocks. This may be the rationale for the lower accuracy in the subsequent application of the data-driven model for the determination of the bending angle at the yellow filter block.

In general, a comparison of the four images enables a simple and fast location of the bending position of the waveguide sensor for a particular state of waveguide bending. The sensor has good repeatability despite some losses and unexpected light-intensity coupling during its use. Based on the data-driven model for signal identification, using this approach to sensing is still feasible.

### 3.2. Random Multi-Bending Sensing

**Figure 9:**
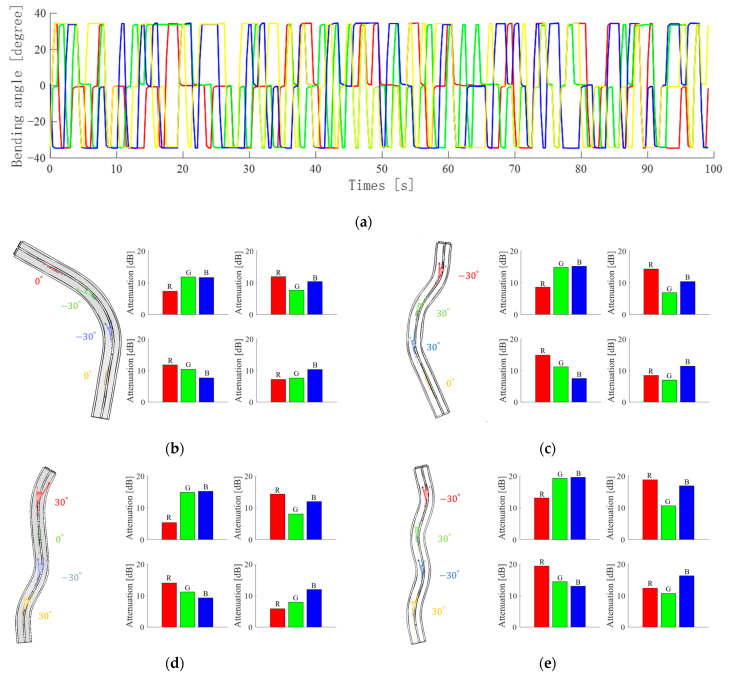
(**a**) Random bending (**b**–**e**) Bending states and the variations of light attenuations obtained based on red, green, blue, and yellow injected lights.

When multiple filter block positions are bent at the same time, light intensities perceived by the color detectors will be significantly different. Three random combinations of waveguide bending to −30 degrees, 0 degrees, or 30 degrees are performed and recorded, as shown in Figure 9a. The x-axis is the time while the y-axis represents the bending angle, and the red, green, blue, and yellow curves represent the positions of the four-color filter blocks on the waveguide sensor, respectively. From these, four typical bending combinations are selected and analyzed and explained one by one, as in Figure 9b–e. In the figures, the left side shows a three-dimensional model diagram of the bending position and angle of the waveguide, which can visualize the state of the waveguide at that time. The four sets of light intensity loss histograms on the right side of the figures represent the results obtained when four different colors of lights are transmitted in the waveguide ${WG}_{a}$. When the waveguides are in a straight state, the light intensity is collected and used as a reference standard for the calculation of light attenuation.

The bending states of the four figures are different. However, there is still a significant amount of bending loss, despite the fact that the cladding is manufactured based on the total reflection principle. Comparing the bending states of the four figures, the fewer the number of bends the waveguides have, the lower the loss is. 

As Figure 9b shows, the first bar graph represents the result of the incident red light. The lower attenuation of red light than green and blue light is due to the coupling of red light into the waveguide ${WG}_{b}$ during transmission. In contrast, on the second and third bar graphs, the incident lights are green and blue, respectively. Despite the bending of the waveguide at both locations, referring to Figure 6c and Figure 7d, bending to the negative direction does not lead to a heavy reduction of light attenuation. In the fourth bar graph, all three attenuations of lights are reduced because the yellow injected light is composed of red and green lights, and the green light contains blue light, as seen in Table 1.

Moreover, the overall light intensity attenuation in Figure 9c rises because there are three bends in the waveguide sensor, including −30 degrees at the red filter block, 30 degrees in green, and 30 degrees in blue. Figure 9d also shows three bends of waveguides, 30 degrees for the red filter block, −30 degrees for the blue, and 30 degrees for the yellow. However, the differences in bending position and direction lead to differences in their light intensity attenuation results. For example, since the green filter block in Figure 9c is bent to 30 degrees while the green filter block in Figure 9d is kept straight, more green light is able to be coupled into the waveguide ${WG}_{b}$ that is bent to the positive direction. Therefore, when the light source contains green light, the green light attenuation is lower for waveguides bent at 30 degrees, as in the second and fourth bar graphs in the figures. Similarly, in Figure 9e, four bends in the waveguide result in greater attenuation, but the angle and direction of the bends cause differences in attenuation in different colors of light and are shown on the four bar graphs.

## 4. Application and Discussion

**Figure 10:**
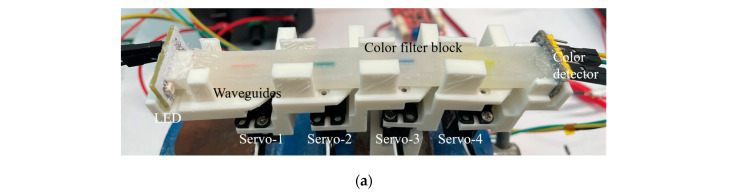

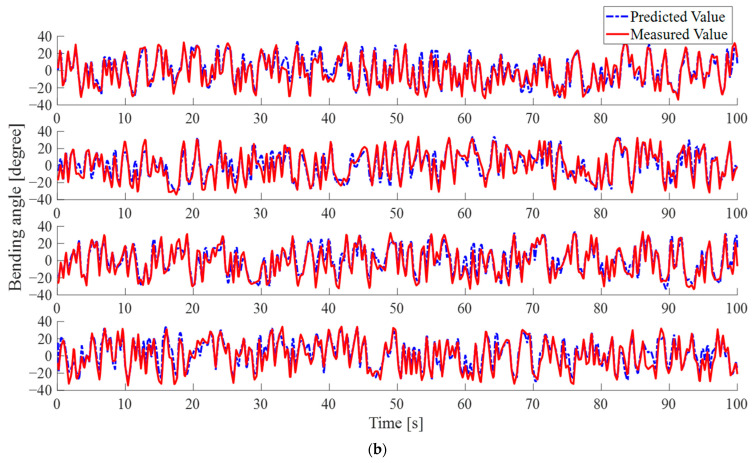
(**a**) Photograph of a waveguide sensor on a bionic multi-joint; (**b**) Measured and predicted values of random bending of the waveguide sensor in four positions over 100 s.

After the analysis of the specific bending state of the waveguide and the attenuation of lights in the waveguides, it provides a qualitative description of the principles and correlations. During the modeling sensing of bending experiments of bionic multi-joints, the bending angles of the waveguide sensor driven randomly by high-precision micro servos are recorded as modeling labels. At the same time, the light intensity variations read from the color detector are recorded as modeling data. The experimental setup is shown in Figure 10a. In addition, the training data contain 20,000 sets of values, which is 5000 random bending states, because there are four sets of values for every bending state. The collection of training data costs about 500 s. Moreover, the test data contain 4000 sets of values, which is 1000 random bending states, and it costs 100 s. The sensing model was trained and tested by times series neural network based on MATLAB R2023 a as applied before [5]. Because the variations of output light intensity are directly related to the bending angles of the optical waveguide, and the signal is sequential in time. 60-layer size is selected for the model, and it costs over 3 h to train the model. When the number of training epochs reaches about 370, the model starts to converge. The trained model can predict the angle of the corresponding waveguide bending based on the light intensity variation from the test data set, which is called the model prediction value. The difference between modeling and enumeration methods lies here. For the enumeration method, if the bending accuracy of the waveguide is designed to be 1 degree, then a waveguide sensor with four bending degrees of freedom requires 12,960,000 bending states. However, the neural network-based modeling approach requires much less data because the neural network-based algorithm is able to take features from the data and make predictions about the bending angle.

Figure 10b shows the predicted and measured values from the four bending positions. The blue dashed lines represent predicted bending angles, and the red solid lines are measured bending angles. It can be found that the predicted values can go well with the measured values.

The correlation coefficient is commonly used in machine learning regression models to express the relationship between predicted and measured values [25,26]. In order to quantify the results of the experiment, the correlation coefficient is chosen to describe the above experimental results and is formulated as follows [27]:

(10) $$\rho (X,Y)=\frac{cov(X,Y)}{\sigma_{X}\sigma_{Y}}$$

In which, cov is the covariance of X and Y, $\sigma_{X}$ is the standard deviation of X, and $\sigma_{Y}$ is the standard deviation of Y. As a result, the correlation coefficients between the two sets of values are listed in Table 2. The correlation coefficients of the measured and predicted values of random bending of the waveguide sensor in 100 s are 0.9211, 0.9106, 0.9219, and 0.9046. Among these, the correlation coefficient of bending at the yellow filter block is relatively low because of the mixed color lights. Besides, the total correlation coefficient is 0.9134, which is better than [5] and meets basic engineering application requirements [28].

**Figure 11:**
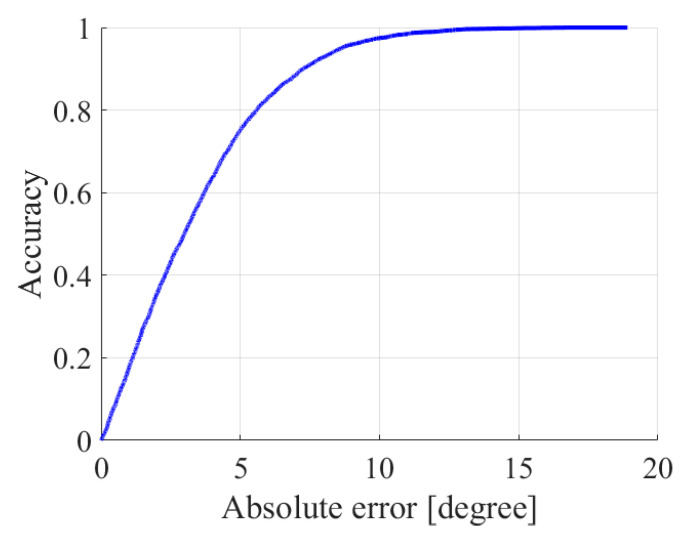
Angle absolute error confidence accuracy curve.

The correlation coefficient enables us to express the relationship between measured and predicted values, but it is difficult to evaluate the accuracy of the data-driven model in a comprehensive and intuitive way. Therefore, the error confidence accuracy curve is presented according to the confidence interval [29]. Figure 11 shows the curve, which is the result obtained by summing up all the detected points within 100 s. The x-axis indicates the absolute value of the error between the predicted and measured results. The y-axis represents the accuracy by the percentage, a point on the curve indicates its percentage when the absolute error is less than or equal to the x-value of the point as an accuracy of its y-value.

## 5. Summary and Conclusions

In this paper, a distributed sensing implementation approach of a fully flexible optical waveguide sensor based on WTDM is proposed and theoretically verified by detailed calculations and derivations. Due to the limitations of the device accuracy of flexible waveguides and their associated manufacturing processes, we combined color filter blocks into the optical waveguide to increase the sensitivity of color light and described and explained in detail the correlation between the bending of the waveguide and the light intensity change of the color. Finally, combined with a data-driven model, the flexible optical waveguide sensor is used for shape sensing of bionic multi-joints, and the coefficient correlation between measured values and predicted values achieved 0.9134. To better illustrate the model, the absolute error confidence accuracy curve is specially presented to describe the accuracy of data-driven models under different errors.

In the future, traditional optical fibers can no longer meet the requirements of flexible bionic robots, while exploring flexible materials and fabrication processes applied to optical waveguides is an important research direction. In addition, the combination of data-driven models to achieve comprehensive and accurate sensor data analysis and demodulation is also a key technology for bionic sensor miniaturization and intelligentization. Simple-structure and stretchable optical shape sensors have a wide range of applications in human-machine interaction, soft robotics, and other fields.

## Figures and Tables

**Table 1:** The color proportion of lights emitted from WS2812 LED.

Color Proportion	Red	Green	Blue
Red light	82%	8%	10%
Green light	7%	66%	27%
Blue light	5%	30%	65%
Yellow light	45%	37%	18%

**Table 2:** Correlation coefficient between actual and predicted values of bending angle of waveguide sensor.

Bending Location	Correlation Coefficient	Total Correlation Coefficient
Bend 1 (R)	0.9211	0.9134
Bend 2 (G)	0.9106	
Bend 3 (B)	0.9219	
Bend 4 (Y)	0.9046	

## Data Availability

Data underlying the results presented in this paper are not publicly available at this time but may be obtained from the authors upon reasonable request.
